# Assessment of the acceptability and usability of an integrated HIV–TB digital portal at anti-retroviral therapy centres in Odisha, India

**DOI:** 10.5588/pha.26.0019

**Published:** 2026-08-24

**Authors:** A. Patnaik, T. Bhatnagar, M. Das, A.K. Singh, N. Mishra, D.S. Arvind, P.K. Siya, A. Pradhan, P. Kumar, B. Patnaik, S.K. Panda, S. Sahu, H. Das, S. Pattanayak

**Affiliations:** 1 Regional Institute for HIV Surveillance & Epidemiology, All India Institute of Medical Sciences, Bhubaneswar, India;; 2 ICMR School of Public Health, ICMR National Institute of Epidemiology (ICMR-NIE), Chennai, India;; 3 FIND, Delhi, India;; 4 Department of Community Medicine and Family Medicine, All India Institute of Medical Sciences, Bhubaneswar, India;; 5 Orissa State AIDS Control Society (OSACS), Bhubaneswar, India;; 6 Department of Health and Family Welfare, Government of Odisha, Bhubaneswar, India;; 7 Surveillance & Epidemiology (Strategic Information) Division, National AIDS Control Organization, New Delhi, India;; 8 WHO State HQ NTEP Consultant, Bhubaneswar, Odisha, India;; 9 Department of Community Medicine, SCB Medical College and Hospital, Cuttack, India;; 10 Department of Health Research, Kalinga Institute of Medical Sciences, Bhubaneswar, India.

**Keywords:** tuberculosis, health information systems, TB–HIV co-infection, health personnel, M-PHISOR

## Abstract

**BACKGROUND:**

In India, health-facility staff at anti-retroviral therapy (ART) centres enter the tuberculosis (TB) status of people living with HIV (PLHIV) in *Ni-kshay* (a TB programme portal) and additionally in an AIDS control programme digital portal, Strengthening Overall Care for HIV Beneficiaries (SOCH).

**OBJECTIVE:**

To determine acceptability and usability of SOCH among health-facility staff for entering TB status of PLHIV in ART centres of Odisha, an Eastern Indian state.

**DESIGN:**

We performed a cross-sectional study from June 2025 to January 2026 in 17 ART centres of Odisha. Acceptability was assessed using the Unified Theory of Acceptance and Use of Technology (UTAUT) model and usability with the System Usability Scale (SUS).

**RESULTS:**

Among 33 staff, median (interquartile range [IQR]) individual scores for four constructs (maximum score: 20) ranged between 15 (14–16) and 16 (12–17). Overall median (IQR) SUS score was 42.5 (31.2–57.5). Lack of training, technical faults, and entering same information in SOCH and *Ni-kshay* portals were reported as reasons for inadequate usage.

**CONCLUSION:**

In Odisha, SOCH was acceptable, albeit with low usability. Periodic training of staff, technical audit of SOCH, and enabling interoperability between HIV and TB portals may improve reporting of HIV–TB care cascade.

In India, digital platforms are routinely used in public health programmes for data capture, reporting, and monitoring. This reduces delays in reporting, supportive supervision, and timely decision making.^1^ However, the benefit from digital platforms depends not only on the software but also on uncomplicated and consistent use in routine programmatic settings.^2^ To understand the performance of digital platforms, the concept of acceptability and usability is important. Acceptability refers to perception and response of users to technology, that is, whether they consider it appropriate, satisfactory, and workable in their setting.^3^ Usability refers to effectiveness, efficiency, and satisfaction of users for completing tasks using technology.^4^ Studies have shown that ease of use, technical quality, and perceived usefulness strongly influence user experience and continued use.^5,6^

Since 2020, under the National AIDS and STD Control Programme (NACP), the portal Strengthening Overall Care for HIV Beneficiaries (SOCH) is used for HIV service documentation and includes TB-related fields for people living with HIV (PLHIV).^7^ Tuberculosis (TB) case notification, diagnosis, treatment, and outcomes are monitored under the National Tuberculosis Elimination Programme (NTEP), through another digital portal – *Ni-kshay*.^8^ HIV–TB-related services are documented in both portals simultaneously. PLHIV are at 20 times higher risk of developing TB than general population.^8^ Timely initiation of treatment is key to reduce morbidity and mortality among PLHIV.^9^ For this, timely documentation of TB diagnosis and treatment is essential. With ∼12.7 million people with presumed TB examined in India, and over 21,000 TB patients with known HIV status, consistent recording is important to monitor service coverage.^7^ Missing or delayed entries may obscure gaps in TB screening, treatment initiation, and follow-up among PLHIV. This may contribute in weakening of monitoring efforts of TB and HIV programmes.^5,7,8^ Existing literature on PLHIV in India are focused on clinical and behavioural factors associated with treatment adherence, clinical profile of patients admitted in hospitals or patients attending ICTC.^10–13^

As per our earlier operational research on the TB care cascade among adult PLHIV registered in anti-retroviral therapy (ART) centres in Odisha, an eastern state in India, although a high proportion were screened for TB, the TB screening status was available in the SOCH portal only for one fifth of registered PLHIV, raising concerns about the usage of the portal.^14^ To the best of our knowledge, no studies have been conducted till date assessing acceptability and usability of the SOCH portal. In this context, we planned this study with the objective of determining the acceptability and usability of the SOCH portal among health-facility staffs in ART centres of Odisha.

## METHODS

We performed a cross-sectional study between June 2025 and January 2026. We included all 20 ART centres where the SOCH portal had been implemented before the initiation of study. In these ART centres, all the health-facility staffs involved in entering TB-related information of PLHIV in the SOCH portal, with a minimum work experience of 3 months in the ART centre, were included in the study. One ART centre was not using the SOCH portal due to paucity of human resources. In two other centres, none of the eligible staff consented for the study. Therefore, 33 health-facility staff from 17 ART centres (care coordinators, counsellors, pharmacists, nursing staffs, and data managers) were included in the study.

### Study setting

TB–HIV services for PLHIV in Odisha are delivered through the Orissa State AIDS Control Society. The ART centres are responsible for creation and maintenance of patient records and entering data into the SOCH portal.^15^ All information is recorded in hard copies, that is, a green book and white card, and is entered in *Ni-kshay* and SOCH portals. Data are entered by different cadres including care coordinators, counsellors, pharmacists, and nursing staffs and verified by the nodal officers with support from medical officers-in-charge and data managers.^16^ Real-time data entries are needed as the portal only accepted current date for TB diagnosis or treatment. The TB-related variables entered in SOCH and *Ni-kshay* are mentioned in Supplementary Data S1.

### Data collection

We used a semi-structured, paper-based, interviewer-administered questionnaire in local vernacular language *(Odia)* for data collection. Information collected on health-facility staffs characteristics included age, gender, designation, employment type, working experience in ART centre, and status of receiving SOCH portal training.

We used the Unified Theory of Acceptance and Use of Technology (UTAUT) model developed by Venkatesh et al. for assessing acceptability of the SOCH portal.^17^ It consisted of four constructs, that is, ‘performance expectancy’, ‘effort expectancy’, ‘social influence’, and ‘facilitating conditions’ **(**Supplementary Data S2). Each construct had four items. Each item in the construct was a statement with a five-point Likert scale response ranging from 1 = strongly disagree to 5 = strongly agree. We used the System Usability Scale (SUS) to determine the usability of the SOCH portal. It contained 10 items with responses in a five-point Likert scale ranging from 0 = strongly disagree to 4 = strongly agree.^18^ Those who scored more than 3 in SUS items 2, 4, 6, 8, and 10 were additionally asked for the reasons for inadequate use of the SOCH portal. Items in the original UTAUT scale and SUS were contextualised to reflect the use of the SOCH portal for entering TB-related information among PLHIV, such that the meaning of the items remained unchanged. Linguistic validation of the tool was done. The tools were carefully reviewed by experts from the ICMR National Institute of Epidemiology (ICMR-NIE), Chennai, and AIIMS Bhubaneswar (Supplementary Data S3).

### Statistical analysis

Data were single-entered, cleaned, and coded in MS Excel and exported to SPSS version 26.0 for analysis. Health-facility staff characteristics were described in frequency and percentages. Designation of health-facility staff was categorised into three groups – care coordinators, nursing staffs, and others. Others included counsellors, pharmacists, and data managers. We calculated median and interquartile range (IQR) for the UTAUT scale and SUS. In the UTAUT scale, the scores ranged from 4 to 20. The higher the score in each construct, higher was the acceptability among health-facility staffs. The SUS gave a single number indicating a composite measure of overall usability of the SOCH portal with minimum score of 0 and maximum 100. SUS scores were categorised into three groups, that is, ‘acceptable’ (>71), ‘marginally acceptable’ (between 51 and 71), and ‘not acceptable’ (<51).^19^ Median (IQR) scores for UTAUT, SUS, and categories of SUS score were also analysed by age, gender, designation, employment type, and training on SOCH portal. We captured the reasons for inadequate use of the SOCH portal as verbatim of 21 (6 care coordinators, 10 nursing staffs, and 5 others, that is, counsellors, lab technicians, and data managers) health-facility staffs. We manually coded the reasons and prepared a word cloud using an online tool (https://www.freewordcloudgenerator.com/).

### Ethical statement

We received approval of the study protocol from the Institutional Human Ethics Committee of ICMR-NIE, Chennai (NIE/IHEC/A/202506-21), Institutional Ethics Committee, AIIMS Bhubaneswar (T/EMF/CM&FM/2025-26/94), and Data Management Committee of Orissa State AIDS Control Society. We obtained written informed consent from each participant.

## RESULTS

The median (IQR) age of health-facility staff was 38 years (30.5–43.5). Out of the 33 health-facility staffs, 17 (52%) were females, 25 (76%) were contractual employees, and 15 (46%) were nursing staffs. Five (15%) health-facility staffs received training on SOCH portal within 1 year of the study. Another 17 (52%) never received training, including 8 (100%) care coordinators and 9 (60%) nursing staffs. Moreover, 14 (56%) of the 25 contractual health-facility staffs and 3 (38%) of the 8 regular employees never received training.

### Acceptability of SOCH portal among health-facility staffs

The median (IQR) score for the ‘performance expectancy’ construct was 15 (14–16). There was no difference in the scores by age, gender, designation, employment type, and being trained in using the SOCH portal. The median (IQR) ‘effort expectancy’ construct score was 16 (16–19). The ‘effort expectancy’ construct score (median [IQR]) among those trained in SOCH portal usage within 1 year (20 [19.5–20]) was higher compared to those who were trained before 1 year (16 [14–18]) or were never trained (16 [16–17]). The overall median (IQR) ‘social influence’ construct score was 16 (12–17). The ‘social influence’ construct score was 16 (13–18) for nursing staffs and 14 (12–17) for those who received training more than 1 year ago (Table 1). Overall median (IQR) construct score for ‘facilitating conditions’ was 16 (14–17). The ‘facilitating conditions’ construct score was 14 (14–16) for nursing staffs and 17 (16–18.7) for care coordinators. This score was 14 (14–15.7) and 16 (14–18.5) for regular and contractual employees, respectively (Table 1).

**TABLE 1.** Acceptability score for UTAUT constructs by characteristics of health-facility staff, ART centres, Odisha, India, 2026 (N = 33).

End-user characteristics (n)	Median (IQR) of acceptability score			
	Performance expectancy	Effort expectancy	Social influence	Facilitating conditions
Overall	15 (14–16)	16 (16–19)	16 (12–17)	16 (14–17)
Age (in years)				
Below 38**^A^** (n = 16)	15 (14–16)	16 (16–18)	16 (12–17)	15 (14–16)
38 and above (n = 17)	16 (14–16)	16 (15–20)	14 (12–17)	16 (14–18)
Gender				
Men (n = 16)	15.5 (14–16)	16.5 (15.2–20)	13.5 (12–17)	16 (14.2–19.7)
Women (n = 17)	15 (14–16)	16 (16–18)	16 (13.5–18)	15 (14–16)
Designation				
Nursing staff (n = 15)	15 (14–16)	16 (16–18)	16 (13–18)	14 (14–16)
Care coordinator (n = 08)	15.5 (14.2–18.2)	16.5 (16–19.7)	16 (12.5–18.5)	17 (16–18.7)
Others^B^ (n = 10)	15.5 (12.7–16)	17 (14.7–20)	12.5 (11.7–17)	15.5 (14.7–20)
Type of employment				
Regular (n = 08)	15 (13.2–16)	16.5 (14.5–19)	15.5 (13.2–18)	14 (14–15.7)
Contractual (n = 25)	15 (14–16)	16 (16–19.5)	16 (12–17)	16 (14–18.5)
Last training received on SOCH				
Within 1 year (n = 05)	16 (13–18)	20 (19.5–20)	18 (10–19.5)	16 (14.5–20)
More than 1 year ago (n = 11)	15 (13–16)	16 (14–18)	14 (12–17)	15 (14–16)
Never received (n = 17)	15 (14–16)	16 (16–17)	16 (12–16.5)	16 (14–17)

UTAUT = Unified Theory of Acceptance and Use of Technology; ART = anti-retroviral therapy; SOCH = Strengthening Overall Care for HIV Beneficiaries.

A Median age of end-users.

B Data managers (n = 05), counsellors (n = 04), and pharmacist (n = 01).

### Usability of SOCH portal among health-facility staffs

The overall median (IQR) SUS score was 42.5 (31.2–57.5). The SUS score was rated as ‘not acceptable’ for 11 (52.4%) nursing staffs, 16 (76.2%) contractual health-facility staffs, and 12 (57.1%) staffs who never received training. None reported ‘acceptable’ score. The SUS score was rated as ‘marginally acceptable’ for five (41.7%) other health-facility staff like counsellors, pharmacists, and data managers. Three (25.0%) who received training on SOCH portal within 1 year rated the score as ‘marginally acceptable’ while 12 (57.1%) who never received training on SOCH portal rated the score as ‘unacceptable’ (Table 2).

**TABLE 2.** System Usability Scale (SUS) scores and categorisation by characteristics of health-facility staff, ART centres, Odisha, India, 2026 (N = 33).

End-user characteristics (n)	SUS score		
	Median (IQR) scores	‘Marginally acceptable’ (N = 12)	‘Not acceptable’ (N = 21)
Age (in years)			
Below 38**^A^** (16)	45.0 (35.0–52.5)	06 (50.0)	10 (47.6)
38 and above (17)	42.5 (21.2–57.5)	06 (50.0)	11 (52.4)
Gender			
Men (16)	42.5 (15.6–57.5)	06 (50.0)	10 (47.6)
Women (17)	42.5 (35.0–55.0)	06 (50.0)	11 (52.4)
Designation			
Nursing staff (15)	42.5 (35.0–52.5)	04 (33.3)	11 (52.4)
Care coordinator (08)	37.5 (16.2–61.8)	03 (25.0)	05 (23.8)
Others^B^ (10)	48.7 (16.8–58.7)	05 (41.7)	05 (23.8)
Type of employment			
Regular (08)	42.5 (35.6–56.2)	03 (25.0)	05 (23.8)
Contractual (25)	45.0 (28.7–57.5)	09 (75.0)	16 (76.2)
Last training received on SOCH			
Within 1 year (05)	52.5 (22.5–60.0)	03 (25.0)	02 (9.5)
More than 1 year ago (11)	42.5 (35.0–57.5)	04 (33.3)	07 (33.3)
Never received (17)	40.0 (28.7–55.0)	5 (41.7)	12 (57.1)

ART = anti-retroviral therapy; SOCH = Strengthening Overall Care for HIV Beneficiaries; IQR = interquartile range.

A Median age of end-users.

B Data managers (n = 05), counsellors (n = 04), and pharmacist (n = 01).

### Reasons for inadequate usage of SOCH portal

Nursing staffs and care coordinators reported not being trained for entering TB-related data in the SOCH portal. They were either oriented by data managers who were trained in portal use or by other existing users. Nursing staffs reported not using computers regularly in their workplace and found entering information in the portal as difficult. They expressed fear of making mistakes and were dependent on data managers for data entry. Care coordinators reported being unsure about TB-data fields in the portal, as they had not been trained on TB terminologies before being assigned the data entry responsibilities. Health-facility staff were overwhelmed with the need for entering the same information in SOCH and *Ni-kshay* portals simultaneously. Data managers mentioned that they had to enter data on behalf of other assigned staff in their absence. Health-facility staff reported technical challenges in the SOCH portal like slow server response, frequent automatic logouts, refreshed pages post–data submission, and occasional loss of entries, which required re-entering the information. In addition, health-facility staff mentioned that TB information did not match with the current programme requirements (e.g., ‘four-symptom screening’ is recorded in the portal while ‘10-symptom screening’ is practised in routine programmatic settings). The technical issues along with continuous patient flow during peak hours made real-time entry in the portal difficult for them (see Figure).

**FIGURE.**
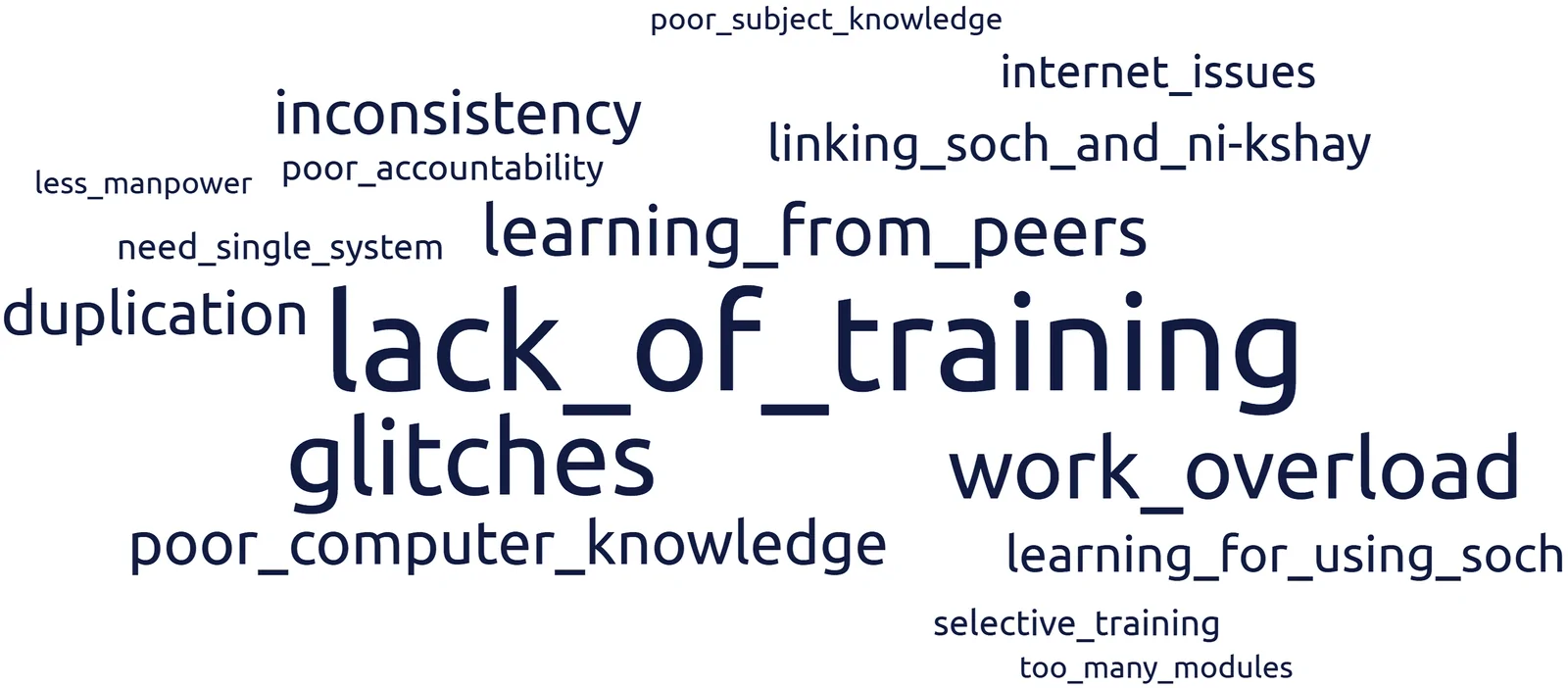
Reasons for inadequate usage of SOCH portal among health-facility staffs, ART centres, Odisha, India, 2026. ART = anti-retroviral therapy; SOCH = Strengthening Overall Care for HIV Beneficiaries.

## DISCUSSION

The ART centre staffs in Odisha, India, found the digital portal of HIV–TB collaborative services (the SOCH portal) to be acceptable but with challenges in its usability. Lack of training, duplication of work, and technical challenges in the portal were common reasons for inadequate use of the digital portal.

Acceptability echoes the emotions, values, and coping mechanisms of health-facility staffs towards a digital portal.^3,20^ In our study, majority of health-facility staff either did not receive training on SOCH portal use or were trained more than a year ago. This lowered their confidence levels and increased dependence on peers in understanding of the SOCH portal. Studies have reported high acceptance for using digital portals in users with defined data entry roles.^21,22^ In this study, acceptability was low among those with multiple responsibilities of clinical service delivery and data entry in digital portals. Lack of training and unavailability of standardised workplan may lead to missing data in digital portals and sub-optimal monitoring of HIV–TB cascade.^17,23,24^ Therefore, we recommend cadre-specific training and supportive supervision with feedback and peer champions model to strengthen acceptability of the SOCH portal.

Usability of a digital tool is often influenced by technical issues like slow operating system and unstable/hard-to-access applications during routine work.^5,18^ In our study, technical issues like automatic logout from portal and error in data upload were common challenges. This might have led to fragmented documentation of HIV–TB information that was observed in our previous study.^14^ Multiple digital portals for different national health programmes often function in parallel, and limited interoperability among them may create duplication of work and reduce usefulness of routine data.^2^ This is especially important for HIV–TB, wherein parallel data entry, workload pressures, and technical issues can affect the completeness and consistency of reporting.^5,9^ The NACP may prioritise improving server connectivity, provision of helpdesk support, aligning platform with recent TB guidelines, and organising periodic technological audits for the SOCH portal.

Although the TB services were provided to PLHIV under HIV–TB collaborative framework, the TB information of PLHIV had to be recorded in two digital portals (SOCH and *Ni-kshay*). Health-facility staff reported duplicate entries in these portals that increased their workload, delayed real-time entry, and increased the risk of data inconsistencies, in line with other studies.^2,5^ The national HIV and TB programmes may prioritise interoperability between digital portals (SOCH and *Ni-kshay*) and routine data reconciliation audits, while at the same time ensuring data confidentiality.^2,5,23,25^ A technical advisory committee may be formed at national and state level to prepare the step-wise algorithms and guidelines for data exchange and data sharing mechanism between SOCH and *Ni-kshay* portals. Once these platforms have been aligned for data entry in one portal and real-time data visualisation in another portal (using a unique identifier), feedback must be received from the health-facility staffs involved in both these portals, for amendments. This will help digital portals in supporting routine HIV–TB integrated services, rather than competing with it.^5,23,26^ For optimal functioning and usage of portals, orientations of the health-facility staffs on the portals at regular intervals and rationalisation of workload must be ensured.^25^

Our study used a robust design and reported findings from all ART centres of Odisha, India. The response rate for our study was high. Our state-wide results may contribute to similar review of portals at national level and thereby support in improvement of digital portal integration for quality monitoring of PLHIV with TB. We have few limitations in our study. First, psychometric validation of the acceptability and usability scales were not carried out. Second, the acceptability scores could be overestimated as the interviewer could be perceived to be linked to national programme influencing the responses by the ART staff. However, to minimise bias, we used a standardised script, assured confidentiality, and explicitly stated that responses would not affect their work appraisal. Third, we did not verify the reported training status of health-facility staff from institutional records. However, there is no reason to doubt the veracity of the same. Fourth, we did not collect information about health-facility staff’s experience of data entry in the digital portals. The slowness of the portal was self-reported and could not be verified by the researchers. Therefore, we could not assess if usability score difference was influenced by staff’s baseline familiarity with digital platforms.

## CONCLUSION

In the ART centres of the eastern state of Odisha in India, the digital portal for reporting TB care among PLHIV was generally acceptable to the health-facility staff, but its usability was limited by lack of training, technical challenges, and duplicate entry across SOCH and *Ni-kshay* portals. Strengthening training activities, providing supportive supervision, and enabling interoperability between both portals can improve data quality and HIV–TB cascade monitoring. A joint assessment of the digital portals by the national AIDS and TB programmes across the country is warranted.

## Supplementary Material
