# Supplementary material for: Assessment of the acceptability and usability of an integrated HIV–TB digital portal at anti-retroviral therapy centres in Odisha, India
Source: Public Health Action. 2026 Aug 24;16(3):172–6. doi: 10.5588/pha.26.0019 (PMC13502502; doi:10.5588/pha.26.0019)
Supplement: Supplementary file 1 [file pha26-0019_supplementarydata1.pdf]

SUPPLEMENTARY FILES

Supplementary file 1: Tuberculosis variables entered in Ni-kshay and SOCH portal

***Ni-kshay* portal**	**SOCH portal**
Diagnosing Facility State	4S Screening
Diagnosing Facility District	IPT status
Diagnosing Facility TBU	Referred for TB testing
Diagnosing Facility PHI	TB Diagnosis
Diagnosing Facility PHI Type	TB treatment status
Diagnosing Facility PHI ID	Date of TB Status change
Diagnosis Date	RIF Resistance
Enrollment Date	
Episode ID	
Old Nikshay ID	
Patient ID	
Patient Status	
Age	
Gender	
Weight	
Key Population	
HIV Status	
Date of ART Initiation	
Diabetes Status	
Basis of Diagnosis Test Name	
Basis of Diagnosis Final Interpretation	
Microbiologically Confirmed	
Date of TB Treatment Initiation	
Type of Case	
PMDT Regimen Type	
Site of Disease	
EP Site	
Treatment Outcome	

Treatment Outcome Date	
Follow-up Done Count	
Contact Tracing Done	
UDST Done	
End of IP SM Date of Report	
End of IP Lab ID	
End of IP Final Interpretation	
End of CP SM Date of Report	
End of CP Lab ID	
End of CP Final Interpretation	
Initial mWRD Offered	
Valid result for Rif available via DRT	
Rif Resistance Detected	
Rif resistance not detected	
Valid Test Result available for Isoniazid via DRT	
INH Resistance Detected	
INH resistance not detected	
Valid Test Result available for FQ susceptibility	
FQ Resistant	
FQ Sensitive	
RR-TB excluding Pre-XDR and XDR-TB	
RR-TB with Pre-XDR or XDR-TB	
Rif resistant with INH resistance detected	
Rif resistant with INH resistance not detected	
Rif resistant not detected with INH resistance not detected	
Rif resistant not detected with INH resistance detected	
Valid results for INH available among RR	
Valid results for INH available among RS	

Valid results for FQ available among RR	
FQ resistance detected among RR (Pre-XDR-TB)	
Pre-XDR-TB with valid DST results for Lzd or Bdq available	
Pre-XDR-TB with Lzd and Bdq resistance (XDR-TB)	
Rif Resistant cases with FQ resistance and resistance for LZD or resistance for BDQ	
Pre-XDR-TB with Lzd resistance or Bdq sensitive(XDR-TB)	
Pre-XDR-TB with Lzd resistance or Bdq unknown(XDR-TB)	
Pre-XDR-TB with Lzd sensitive or Bdq resistance(XDR-TB)	
Pre-XDR-TB with Lzd sensitive or Bdq sensitive	
Pre-XDR-TB with Lzd sensitive or Bdq unknown	
Pre-XDR-TB with Lzd unknown or Bdq resistance(XDR-TB)	
Pre-XDR-TB with Lzd unknown or Bdq sensitive	
Pre-XDR-TB with Lzd unknown or Bdq unknown	
DR-TB cases without valid DST/DRT result available	

Table 1. Operational definitions for constructs used in the UTAUT* model[17]

**Constructs**	**Operational definition**
Performance expectancy (PE)	Degree to which end-user believes that using the system will help him or her to attain gains in job performance. If an end-user is convinced that the new technology is more efficient and productive, then he/ she will be encouraged to adopt it.
Effort expectancy (EE)	Degree of ease associated with the use of the system. The end - user would like to use the technology because it is simple, easy to access and use.
Social influence (SI)	Degree to which an individual perceives importance of the system through the influence of others like fellow co-workers. The end-users would like to utilize the technology if their co-workers use them.
Facilitating conditions (FC)	Degree to which an individual believes that an organizational and technical infrastructure exists to support use of the technology. This construct is measured by the perception of being able to access required resources and the necessary support needed to use the technology

*: *Unified Theory of Acceptance and Use of Technology*

Supplementary file 3: The Unified Theory of Acceptance and Use of Technology (UTAUT) bi-lingual tool for assessing acceptability of health facility staff in ART centres of Odisha, India

Sl. No.	Items (Acceptability of SOCH portal)	Likert scale from 1 to 5 (Strongly Disagree to Strongly Agree) ପୁରାପୁରି ଅସହମତ → ପୁରାପୁରି ସହମତ
1	I find SOCH portal useful for entering data related to TB<br><br>TB ସମ୍ବନ୍ଧୀୟ ତଥ୍ୟ ଏଣ୍ଟ୍ରି କରିବା ପାଇଁ SOCH ପୋର୍ଟାଲ୍ ଉପଯୋଗୀ ବୋଲି ମୁଁ ମନେ କରେ	
2	Using the SOCH portal enables me to enter data related to TB more quickly<br><br>ମୁଁ SOCH ପୋର୍ଟାଲ୍ ବ୍ୟବହାର କରିଲେ TB ସମ୍ବନ୍ଧୀୟ ତଥ୍ୟ ଶୀଘ୍ର ଏଣ୍ଟ୍ରି କରିପାରୁଛି	
3	Using the SOCH portal increases my productivity<br><br>SOCH ପୋର୍ଟାଲ୍ ବ୍ୟବହାର କରିଲେ ମୋର କାର୍ଯ୍ୟକୁଶଳତା ବୃଦ୍ଧି ପାଉଛି	
4	If I use the SOCH portal, I will increase my chances of getting a raise<br><br>SOCH ପୋର୍ଟାଲ୍ ବ୍ୟବହାର କଲେ ମୋ ପଦୋନ୍ନତିର ସମ୍ଭାବନା ବଢ଼ିପାରେ	
5	My interaction with the SOCH portal is clear and understandable<br><br>SOCH ପୋର୍ଟାଲ୍ ସହିତ ମୋର କାର୍ଯ୍ୟକଳାପ ସ୍ୱଷ୍ଟ ଏବଂ ବୁଝିବାଯୋଗ୍ୟ	
6	It is easy for me to be skillful at using the SOCH portal<br><br>SOCH ପୋର୍ଟାଲ୍ ବ୍ୟବହାରରେ ଦକ୍ଷ ହେବା ମୋ ପାଇଁ ସହଜ	
7	I find the SOCH portal easy to use<br><br>SOCH ପୋର୍ଟାଲ୍ ବ୍ୟବହାର କରିବାକୁ ମୋତେ ସହଜ ଲାଗେ	
8	Learning to operate the SOCH portal is easy for me<br><br>SOCH ପୋର୍ଟାଲ୍ ଚାଲାଇବା ଶିଖିବା ମୋ ପାଇଁ ସହଜ	
9	Using the SOCH portal is a good idea<br><br>SOCH ପୋର୍ଟାଲ୍ ବ୍ୟବହାର କରିବା ଗୋଟିଏ ଭଲ ଧାରଣା	
10	The SOCH portal makes work interesting<br><br>SOCH ପୋର୍ଟାଲ୍ ମୋ କାମକୁ ଆକର୍ଷଣୀୟ କରେ	

11	Working with the SOCH portal is fun<br><br>SOCH ପୋର୍ଟାଲ୍ ସହ କାମ କରିବା ମଜାଦାର	
12	I like working with the SOCH portal<br><br>SOCH ପୋର୍ଟାଲ୍ ସହ କାମ କରିବାକୁ ମୁଁ ପସନ୍ଦ କରେ	
13	People who influence my behaviour think that I should use the SOCH portal<br><br>ଯେମାନେ ମୋ ଆଚରଣକୁ ପ୍ରଭାବିତ କରନ୍ତି, ସେମାନେ SOCH ପୋର୍ଟାଲ୍ ବ୍ୟବହାର କରିବାକୁ ମୋତେ କହନ୍ତି	
14	People who are important to me think that I should use the SOCH portal<br><br>ଯେମାନେ ମୋ ପାଇଁ ଗୁରୁତ୍ୱପୂର୍ଣ୍ଣ, ସେମାନେ SOCH ପୋର୍ଟାଲ୍ ବ୍ୟବହାର କରିବାକୁ ମୋତେ କହନ୍ତି	
15	The senior management of this ART Centre has been helpful in the use of SOCH portal<br><br>ଏହି ART କେନ୍ଦ୍ରର ବରିଷ୍ଠ ପ୍ରଶାସନ SOCH ପୋର୍ଟାଲ୍ ବ୍ୟବହାରରେ ସହାୟକ ହୋଇଛନ୍ତି	
16	In general, the ART Centre has supported the use of the SOCH portal<br><br>ସାଧାରଣତଃ ART କେନ୍ଦ୍ର SOCH ପୋର୍ଟାଲ୍ ବ୍ୟବହାରକୁ ସମର୍ଥନ କରିଛି	
17	I have resources necessary to use the SOCH portal<br><br>SOCH ପୋର୍ଟାଲ୍ ବ୍ୟବହାର ପାଇଁ ଆବଶ୍ୟକ ସମ୍ପଦ ମୋ ପାଖରେ ଅଛି	
18	I have the knowledge necessary to use the SOCH portal<br><br>SOCH ପୋର୍ଟାଲ୍ ବ୍ୟବହାର ପାଇଁ ଆବଶ୍ୟକ ଜ୍ଞାନ ମୋ ପାଖରେ ଅଛି	
19	The SOCH portal is not compatible with other systems I use<br><br>SOCH ପୋର୍ଟାଲ୍ ମୋର ଅନ୍ୟ ସିଷ୍ଟମ୍‌ଗୁଡ଼ିକ ସହ ସୁସଙ୍ଗତ ନୁହେଁ	
20	A specific person (or group) is available for assistance with system difficulties<br><br>ସିଷ୍ଟମ୍ ସମସ୍ୟା ସମୟରେ ସହାୟତା ପାଇଁ ନିର୍ଦ୍ଦିଷ୍ଟ ବ୍ୟକ୍ତି (କିମ୍ବା ଦଳ) ଉପଲବ୍ଧ	
21	I can complete a job or task using the SOCH portal, if there is no one around to tell me what to do as I go<br><br>କେହି ମୋତେ ନିର୍ଦ୍ଦେଶ ନଦେଲେ ମଧ୍ୟ SOCH ପୋର୍ଟାଲ୍ ବ୍ୟବହାର କରି ମୁଁ କାମ ସମାପ୍ତ କରିପାରେ	
22	I can complete a job or task using the SOCH portal, if I can call someone for help if I got stuck	

	ମୁଁ ଅଟକିଗଲେ କାହାକୁ ଫୋନ୍ କରି ସହାୟତା ନେଇ SOCH ପୋର୍ଟାଲ୍ ବ୍ୟବହାର କରି କାମ ସମାପ୍ତ କରିପାରେ	
23	I can complete a job or task using the SOCH portal, if I have a lot of time to complete the job for which the software is provided<br><br>ଯଥେଷ୍ଟ ସମୟ ଥିଲେ SOCH ପୋର୍ଟାଲ୍ ବ୍ୟବହାର କରି କାମ ସମାପ୍ତ କରିପାରେ	
24	I could complete a job or task using the SOCH portal, if I have just the built-in help facility for assistance<br><br>କେବଳ ସିଷ୍ଟମ୍‌ର ଭିତରେ ଥିବା ସହାୟତା ବିକଳ୍ପ ବ୍ୟବହାର କରି SOCH ପୋର୍ଟାଲ୍ ସହ କାମ କରିପାରେ	
25	I feel apprehensive about using the SOCH portal<br><br>SOCH ପୋର୍ଟାଲ୍ ବ୍ୟବହାର କରିବା ବେଳେ ମୋତେ ଆତଙ୍କ ଲାଗେ	
26	It scares me to think that I can lose a lot of information using the SOCH portal by hitting the wrong key<br><br>ଭୁଲ କୀ ଦବାଇଦେଲେ SOCH ପୋର୍ଟାଲ୍‌ରେ ବହୁତ ସୂଚନା ହରାଇଯିବ ବୋଲି ଭାବି ମୋତେ ଭୟ ଲାଗେ।	
27	I hesitate to use the SOCH portal for fear of making mistakes I cannot correct<br><br>ସୁଧାରି ପାରିବି ନାହିଁ ବୋଲି ଭୟରୁ SOCH ପୋର୍ଟାଲ୍ ବ୍ୟବହାର କରିବାରେ ମୁଁ ହେସିଟେଟ୍ କରେ	
28	The SOCH portal is somewhat intimidating to me<br><br>SOCH ପୋର୍ଟାଲ୍ ମୋ ପାଇଁ କିଛି ପରିମାଣରେ ଭୟଜନକ ମନେ ହୁଏ	

Supplementary table 3: System Usability Scale (SUS) bi-lingual tool for assessing usability of SOCH portal among health facility staff at ART centres of Odisha, India

Sl. No.	Questions (System Usability Scale)	Rating from 1 to 5 (Strongly disagree to Strongly agree)<br>ମୂଲ୍ୟାଙ୍କନ: 1 ରୁ 5 (ପୁରାପୁରି ଅସହମତ ରୁ ପୁରାପୁରି ସହମତ)
1	I think that I like to use the SOCH portal frequently for entering TB care information of PLHIV<br><br>ମୋତେ ଲାଗେ ଯେ PLHIV ଙ୍କ TB ଚିକିତ୍ସା ସମ୍ବନ୍ଧୀୟ ସୂଚନା ଏଣ୍ଟ୍ରି କରିବା ପାଇଁ SOCH ପୋର୍ଟାଲ୍‌କୁ ମୁଁ ବାରମ୍ବାର ବ୍ୟବହାର କରିବାକୁ ପସନ୍ଦ କରେ	
2*	I find the SOCH portal unnecessarily complex for recording TB -related information of PLHIV.<br><br>PLHIV ଙ୍କ TB ସମ୍ବନ୍ଧୀୟ ସୂଚନା ରେକର୍ଡ କରିବା ପାଇଁ SOCH ପୋର୍ଟାଲ୍‌କୁ ମୁଁ ଅନାବଶ୍ୟକ ଭାବେ ଜଟିଳ ମନେ କରେ	
3	I think the SOCH portal is easy to use for entering TB related information<br><br>PLHIV ଙ୍କ TB ସମ୍ବନ୍ଧୀୟ ସୂଚନା ଏଣ୍ଟ୍ରି କରିବା ପାଇଁ SOCH ପୋର୍ଟାଲ୍ ବ୍ୟବହାର କରିବା ସହଜ ବୋଲି ମୋତେ ଲାଗେ	
4*	I think I need the support of a technical person to use the SOCH portal effectively for entering data related to TB<br><br>TB ସମ୍ବନ୍ଧୀୟ ତଥ୍ୟ ଏଣ୍ଟ୍ରି କରିବା ପାଇଁ SOCH ପୋର୍ଟାଲ୍‌କୁ ଭଲ ଭାବରେ ବ୍ୟବହାର କରିବାକୁ ମୋତେ ଗୋଟିଏ ଟେକ୍ନିକାଲ୍ ବ୍ୟକ୍ତିଙ୍କ ସହାୟତା ଆବଶ୍ୟକ	
5	I find that various functions in the SOCH portal for entering TB related information is well integrated<br><br>TB ସମ୍ବନ୍ଧୀୟ ସୂଚନା ଏଣ୍ଟ୍ରି ପାଇଁ SOCH ପୋର୍ଟାଲ୍‌ର ବିଭିନ୍ନ କାର୍ଯ୍ୟଗୁଡ଼ିକ ଭଲ ଭାବରେ ସମନ୍ୱିତ ବୋଲି ମୁଁ ମନେ କରେ	
6*	I think there is too much inconsistency in terms of entering TB related information in the SOCH portal.<br><br>SOCH ପୋର୍ଟାଲ୍‌ରେ TB ସମ୍ବନ୍ଧୀୟ ସୂଚନା ଏଣ୍ଟ୍ରି କରିବାରେ ଅଧିକ ଅସଙ୍ଗତି ରହିଛି ବୋଲି ମୋତେ ଲାଗେ	
7	I can imagine that most ART Centre staff have learnt to use the SOCH portal for entering TB related information very quickly	

	ଅଧିକାଂଶ ART କେନ୍ଦ୍ରର କର୍ମଚାରୀମାନେ TB ସମ୍ବନ୍ଧୀୟ ସୂଚନା ଏଣ୍ଟ୍ରି ପାଇଁ SOCH ପୋର୍ଟାଲ୍‌କୁ ଖୁବ ଶୀଘ୍ର ଶିଖିନେଇଛନ୍ତି ବୋଲି ମୁଁ କଳ୍ପନା କରିପାରେ	
8*	I find the SOCH portal very cumbersome to use for entering TB related information<br><br>TB ସମ୍ବନ୍ଧୀୟ ସୂଚନା ଏଣ୍ଟ୍ରି କରିବା ପାଇଁ SOCH ପୋର୍ଟାଲ୍ ବ୍ୟବହାର କରିବା ବହୁତ କଷ୍ଟକର ବୋଲି ମୋତେ ଲାଗେ	
9	I feel very confident using the SOCH portal to document and retrieve TB related information<br><br>TB ସମ୍ବନ୍ଧୀୟ ସୂଚନା ଲେଖାବଦ୍ଧ କରିବା ଏବଂ ଖୋଜିବା ପାଇଁ SOCH ପୋର୍ଟାଲ୍ ବ୍ୟବହାର କରିବାରେ ମୁଁ ବହୁତ ଆତ୍ମବିଶ୍ୱାସୀ ଅନୁଭବ କରେ	
10*	I needed to learn a lot of things before I started using the SOCH portal for entering TB related information<br><br>TB ସମ୍ବନ୍ଧୀୟ ସୂଚନା ଏଣ୍ଟ୍ରି ପାଇଁ SOCH ପୋର୍ଟାଲ୍ ବ୍ୟବହାର କରିବା ପୂର୍ବରୁ ମୋତେ ବହୁତ କଥା ଶିଖିବାକୁ ପଡ଼ିଥିଲା	

***For items 2,4,6,8, and 10, if the respondent scores more than 3, then he/she will be asked the reasons why does he/she feel that way.**

*** ପ୍ରଶ୍ନ 2, 4, 6, 8 ଏବଂ 10 ରେ ଯଦି ମୂଲ୍ୟାଙ୍କନ 3 ରୁ ଅଧିକ ହୁଏ, ତେବେ ଏହା ପାଇଁ କାରଣ ପଚାରାଯିବ।**
